# Process and dynamics of traditional selling wild edible mushrooms in tropical Mexico

**DOI:** 10.1186/1746-4269-2-3

**Published:** 2006-01-05

**Authors:** Felipe Ruán-Soto, Roberto Garibay-Orijel, Joaquín Cifuentes

**Affiliations:** 1 Herbario Eizi Matuda, Escuela de Biología, Universidad de Ciencias y Artes de Chiapas, Libramiento Norte Poniente S/N C.P. 29039, Tuxtla Gutiérrez, Chiapas, México; 2 Facultad de Ciencias, Universidad Nacional Autónoma de México, Apdo. Postal 113-100, Rumania N° 700, Col. Portales, C.P. 03301, México, D.F; 3 Sección de Micología, Herbario FCME, Facultad de Ciencias, Universidad Nacional Autónoma de México, Apdo. Postal 70-181, C.P. 04510, Ciudad Universitaria, México D.F; 4 Interdisciplinary group for Ethnomycology Development, México

## Abstract

**Background:**

More than twelve temperate-inhabitant Mexican ethnic groups are considered to be mycophilic and to have extensive traditional mycological knowledge. In contrast, inhabitants of tropical lands have been studied only superficially and their mycological knowledge is less well known. In this paper, we report the results of an ethnomycological research in markets of a wide area of the Mexican tropics. Our aims were to describe the dynamics related to the traditional selling process of wild mushrooms and to determine the tendencies of informants toward mushrooms (mycophily *vs*. mycophoby).

**Methods:**

We visited 25 markets of 12 different settlements in the states of Oaxaca, Tabasco and Veracruz and collected information by participant observation as well as by 291 non-structured and semi-structured interviews.

**Results:**

Mushroom selling was observed in four towns in Oaxaca and in two in Tabasco. Women represented 81.82% of sellers, while indigenous people (Chinantecos, Chontales, Ch'oles and Zoques) comprised 68.18%. Mushroom commercialization took place in secondary mobile markets and only in peasant stands. Mushroom collectors gather the resource in places with secondary vegetation, farmed areas and cattle fields. Because of land tenure restrictions mushroom sellers did not normally collect mushrooms themselves. In Oaxaca, we observed economic dynamics not based on capitalism, such as exchange, reciprocity and barter.

**Conclusion:**

The sale of some wild edible mushrooms, the large amounts of commercialization of *Schizophyllum commune*, the complicated intermediary process, as well as the insertion of mushrooms into different informal economic practices are all evidence of an existent mycophily in a sector of the population of this region of the Mexican tropics. Among our informants, urban mestizo people were mycophobic, rural mestizo people were non-mycophilic and indigenous people were true mycophilic.

## Resumen

En México más de 12 étnias habitantes de zonas templadas han mostrado una tendencia micofílica y un profundo conocimiento micológico tradicional. Sin embargo los habitantes de zonas tropicales han sido estudiados insuficientemente. En el presente trabajo reportamos los resultados de una investigación etnomicológica en mercados de una amplia zona del trópico mexicano. Los objetivos del estudio fueron: describir las dinámicas relacionadas con la venta de hongos silvestres, así como, indagar cuál es la tendencia de los pobladores hacia los hongos (micofilia *vs*. micofobia). Visitamos 25 mercados en 12 poblaciones de 3 estados (Oaxaca, Tabasco y Veracruz). Llevamos a cabo observación participante y aplicamos 291 entrevistas no estructuradas y semiestructuradas. La venta de hongos se observó en cuatro poblaciones del estado de Oaxaca y en dos de Tabasco. El 81.82% de los vendedores de hongos fueron mujeres y el 68.18% indígenas (Chinantecos, Chontales, Ch'oles y Zoques). La venta de hongos sólo se observó en mercados secundarios de tipo "tianguis" y sólo en puestos campesinos. Los recolectores de hongos extraen el recurso principalmente de zonas con vegetación secundaria, cultivos y potreros. Por restricción de acceso a la tierra los vendedores no son quienes recolectan los hongos. En Oaxaca, se observaron dinámicas económicas distintas a las capitalistas como intercambio, reciprocidad y trueque. La comercialización de algunas especies de hongos comestibles silvestres, los grandes volúmenes de venta de *S. commune*, los complejos procesos de intermediarios, así como la inserción de los hongos en dinámicas económicas informales son evidencias de la micofilia existente en un sector de la población de esta región tropical de México. Entre nuestros informantes, los mestizos urbanos fueron micófobos, los mestizos rurales fueron no micófilos y los indígenas fueron micófilos.

### Palabras clave

Etnomicología tropical, micofilia, micofobia, Planicie costera del Golfo de México, conocimiento micológico tradicional.

## Background

Tropical Ethnomycology has been studied scarcely in the field of Ethnobiology. Perhaps because of the persistent belief that tropical mushrooms are unused and that lowland Mesoamerican and Amazonian people are mycophobic (people that demonstrate aversion towards mushrooms) [*c.f*. [1,2]] or non-mycophilic (people with any special interest in mushrooms, not attraction neither repulsion) [3,4]. However, recent research in the tropics [5-10] shows that this judgment is based on insufficient and skewed information. It is a fact that fungal resources are different in temperate areas than in the tropics, where fleshy mushrooms susceptible of consumption are scarce [5]. Nevertheless, mycophily [*sensu *[11]] points the attitude toward mushrooms, not only on the number of species known, but also on the sympathy and whim to them. Additionally, Goes-Neto & Bandeira [4] define that mycophilic people are those which demonstrate special interest towards fungi, such as an important food or cultural activity item.

Numerous studies have reported mushroom consumption by ethnic groups of several tropical areas. Examples in Africa include: Burundi [12], Cameroon [5], Nigeria [13], Tanzania [14], Zambia [15] and Zaire [16]. In Asia: China [17,18], India [19], Malaysia [6], Papua New Guinea [20] and Thailand [21]. In America: Brazil [1,22], El Salvador [2], Guatemala [23], Mexico [24,7] and Venezuela [8]. Given this evidence, it is inadequate to generalize about the mycophoby or non-mycophily of inhabitants of tropical areas.

From 1964 to 1999, only four papers appeared in *Abstracts of Mycology *that included data on Ethnomycology in tropical markets, namely Mata [25], Singh and Kumar [26], Jones and Whalley [27] and Moreno-Black et al. [28]. Sommerkamp [23] also undertook a study about edible mushrooms in markets of Guatemala where five of 22 studied markets were in tropical areas. She found that out of the 22 mushrooms being sold in total, only four species were sold in tropical markets: *Agaricus campestris*, *Pseudofistulina radicata*, *Favolus brasilensis *(*Favolus tenuiculus*) and *Schizophyllum commune*.

Mexico is the 6^th ^country in the world with the largest number of ethnic groups. This cultural diversity includes 62 ethnic groups [29] speaking 290 dialect variants and representing 13% of the total population [30]. Mushroom consumption is a widespread tradition within these groups that can be traced back to pre-Hispanic times. This is evidenced by the Mayan mushroom-stones from the period of 1000-200 b.C. as well as by mushroom representations in codexes as Vindobonensis, Magliabechi, Florentino and Indigena N° 27. All this supports a clear mycophilic attitude amongst Mesoamerican inhabitants. However, even though the tropics are home to 32% of Mexican ethnic groups and most of its biodiversity, ethnomycological studies are scarce [31]. Mata [25] reported folk taxonomy and nomenclature, morphology and phenology of mushrooms used by Yucatan Mayas. He also described the medicinal use of *Thelephora paraguayensis *and *Geastrum triplex*. Chacón [24] found that *Schizophyllum commune *is the most appreciated mushroom in some areas of Veracruz. In his study he also reported as edibles: *Auricularia fuscosuccinea*, *A. mesenterica*, *Armillaria tabescens*, *Cookeina sulcipes*, *C. tricholoma*, *Hohenbuehelia petaloides*, *Pleurotus ostreatus*, *Panus crinitus*, *Schizophyllum commune*, *S. fasciatum *and *Ustilago maydis*.

The lack of ethnomycological data in most of the Mexican tropics has lead to an uncertainty of its inhabitants as being mycophilic or mycophobic, as well as a lack of knowledge in patterns of mushroom consumption and use.

In Ruan-Soto, et al. [7] we proved that studying markets can be a useful tool when evaluating traditional mycological knowledge (TMK) in a wide and previously unstudied area. We also described the extent of the body of TMK ("corpos" *sensu *[32]) that sellers of wild edibles have in the south Mexican Gulf coastal plain. The most important conclusions we reached in that paper were: i) Mestizos from urban settlements do not normally consume wild mushrooms, mestizos from rural areas consume few wild mushrooms, and indigenous people have a wide TMK and they use and exploit this natural resource the most. ii) Mushroom species with a corky or rubbery consistency, particularly *S. commune*, have high cultural and economic importance. iii) People in the area believe that all terricolous-humicolous mushrooms are toxic. iv) Mushroom gathering takes place in corn fields, secondary vegetation and cattle fields and not in areas where conserved vegetation is present.

The aims of the present work were: i) To describe the practice and dynamics ("praxis" *sensu *[32]) surrounding the wild mushroom selling process. ii) To analyze these dynamics from an Economic Anthropology standpoint, and iii) To inquire if informants are mycophilic, non-mycophilic or mycophobic through the understanding of their attitude toward the mushrooms.

## Methods

### Study area

**Figure 1:**
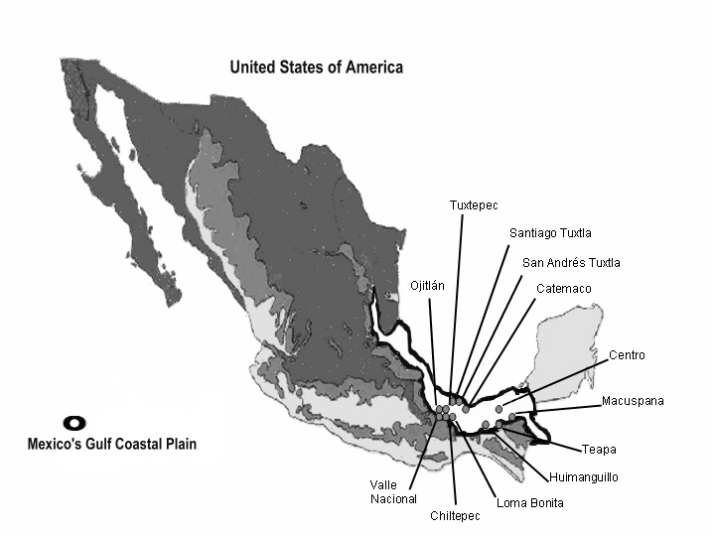
**Mexican Gulf coastal plane and studied sites location. **Modified from Rzedowski (1981).

The work took place in the south Mexican Gulf coastal plain in the settlements of: Tuxtepec, Ojitlán, Chiltepec, Loma Bonita, and Valle Nacional in the state of Oaxaca; Santiago Tuxtla, San Andrés Tuxtla, and Catemaco in the state of Veracruz; and Villahermosa, Macuspana, Teapa, and Huimanguillo in the state of Tabasco (Figure 1). The prevailing original vegetation is tropical rain forest, distributed in plain areas (no more than 100 m.asl). It presents an Af(m) clime in Köppen's clasification; it is humid warm, with annual precipitations above 2000 mm and a temperature variation between 22 and 26°C [33]. Inhabitants of the area include: "Chinantecos" in Oaxaca; "Zoques", "Ch'oles" and "Chontales" in Tabasco; and mestizos in Veracruz. Table 1 shows a summary of ethnic characteristics.

**Table 1:** Ethnic characteristics

Ethnic group	Population	Settlements	Economy	Diet
Chinantecos	133,374	Oaxaca: Tlacoatzintepec, Sochiapan, Ayotzintepec, Usila, Chiltepec, Ojitlán, Jacatepec, Valle Nacional, Lalana, Petlapa, Jocotepec, Quiotepec, Yolox, Comaltepec and Tuxtepec	Agriculture, cattling, hunting and gathering	Corn, beans, chilly, pumpkin, "chayote", wild plants and animals
Ch'oles	161,766	Chiapas: Palenque, Tila, Tumbalá, Salto del agua and Sabanilla. Tabasco: Macuspana	Corn agriculture, coffee, fruits and cattling	Corn, fruits, roots, vegetables and meat
Zoques	51,464	Chiapas: Pichucalco, Reforma, Estación Juárez, Amatán, Ixtacomitán and Tapilula. Tabasco: Huimanguillo and Teapa	Corn agriculture, coffee, wood, cacao and labor	Corn, beans, chilly and wild edibles
Chontales	38,561	Tabasco: Centla, Centro, Jonuta, Nacajuca and Macuspana	Intensive agriculture, cacao, coffee, tobacco, sugar cane, fishing and hunting	Corn, beans, chilly, pumpkin and fish

Chinantec people inhabit the rain forest near Tuxtepec, Oaxaca. They speak chinantec, an offshoot of the otomague linguistic family. Their principal activities are agriculture and cattle raising, but hunting and collection of wild products also play an important role, and this is reflected on their varied diet. Their economy is a mixture of pre-Hispanic and modern practices. They rely on economic practices like exchange, redistribution and reciprocity. Their language has a very complex ethnobotanic taxonomy. In this sense, the Ojitlán Chinantecs are capable of distinguishing between eight different kinds of soil, true evidence of their deep environmental knowledge [34].

Ch'oles inhabit the north of the state of Chiapas and some south Tabasco municipalities. Their language is ch'ol, belonging to the maya-totonac family. Slash-and-burn agriculture is their main economic activity; but lately there is an incease in the amount of cattle raising. To them, every plant and animal has its name, history and place in the origin myths. Moreover, a lot of them have practical or ritual uses [35].

Zoques inhabit in the northwest of Chiapas and the bordering area with Tabasco. Their language is zoque, belonging to maya-totonac family. Their diet is based on products obtained from corn and coffee plantations. Agriculture is their main economic activity; however a considerate amount of them emigrate to cities in search for work [35,36]. This group is characterized by having a profound plant knowledge and a strong dependence on them [37].

Chontales inhabit the centre of Tabasco's wetlands. Their economic activity is varied; they are farmers and fishermen. Agriculture is intensive; reaping two or three crops a year. Their diet is composed of field products, wild products and fish from nearby bodies of water. Oil exploitation has deteriorated the local environment while employing many of the males of the area [35].

The majority of towns near Los Tuxtlas are settlements not older than 50 years, spawned from the agrarian policies implemented by the Mexican government since the 1950's. Many of these immigrants were challenged by a new and unknown environment, lacking the necessary knowledge to manage it adequately [38]. They are primarily extensive cattle raisers and farmers.

### Field work

Market characterization was based on the concepts of: i) "Established market", defined space with fixed infrastructure; ii) "Mobile market", not fixed and can operate all week or just one day ("day local market"); iii) "Peasant stand", sells mainly edible products from peasant farm fields as well as wild products; iv) "Modern stand", sells any product and edible ones come from intermediary chains. Market systems were chosen using Beals [39] market classification, where principal markets and subordinated secondary markets are defined. Exact market location and names can be consulted in [7]. Table 2 shows a summary of characteristics of visited markets.

**Table 2:** Market location and categorization

State	Settlement	Category	Kind of market	N° of interviewees
O	Tuxtepec	P	E	4
O	Tuxtepec	P	E	10
O	Tuxtepec	S	M	13
O	Ojitlán	S	Dlm	5
O	Chiltepec	S	Is	2
O	Valle nacional	S	Dlm	8
O	Loma Bonita	S	E	8
O	Loma Bonita	S	M	11
V	Santiago Tuxtla	S	E	20
V	Santiago Tuxtla	S	M	21
V	San Andrés Tuxtla	P	E	11
V	San Andrés Tuxtla	S	M	23
V	Catemaco	S	E	4
V	Catemaco	S	M	8
T	Villahermosa	P	E	23
T	Villahermosa	P	E	13
T	Villahermosa	S	M	31
T	Teapa	S	E	3
T	Teapa	S	M	23
T	Macuspana	S	E	3
T	Macuspana	S	E	8
T	Macuspana	S	M	12
T	Macuspana	S	M	16
T	Huimanguillo	S	E	7
T	Huimanguillo	S	M	4

In State, O: Oaxaca; V: Veracruz; T: Tabasco. In Category, P: Principal; S: Secondary. In Kind of market, E: Established market; M: Mobile market; Dlm: Day local market; Is: Isolated stands.

We visited 25 markets in 12 settlements of the states of Oaxaca, Veracruz and Tabasco. Field work was done from 2000 to 2002, with six weeks in Oaxaca, five in Veracruz and seven in Tabasco. We interviewed all sellers with modern stands that offered edible products and all sellers with peasant stands. In total, we interviewed 291 people: 172 with modern stands and 119 with peasant stands. From these, 158 were men, 133 were women and 5.84% spoke some indigenous language. We employed the participant observation method and applied non-structured and semi-structured interviews [40]. We used non-structured interviews with all sellers and semi-structured interviews with all sellers of wild mushrooms (Table 3). To describe the gathering process, we applied the participant observation technique, doing gathering trips with informants whenever it was possible.

**Table 3:** Non-structured interview thematic guide and semi-structured interview basic format

Thematic guide	Semi-structured interview basic format*
General information: -Market kind -Market rhythm -Stand organization -Type of product sold -Sellers origin: geographic and ethnic -Products geographical origin -Other sellers activities Ethnomycological information: -Mushrooms species sold -Mushrooms species used but not sold -Gender patterns -Ethnicity of mushrooms sellers -Mushroomers presence -Stand dynamics	-What mushrooms do you sell? -Why do you sell these kinds of mushrooms? -Do you sell other kind of products? -Does anybody sell only mushrooms? -Why you sell mushrooms? -Are you acquainted with someone who knows much about mushrooms? -How many people sell mushrooms here? -Who sells mushrooms, women or men? -Where are mushrooms sellers from? -Do you gather mushrooms? -Do you gather mushrooms alone? -What time do you invest gathering mushrooms? -Do you gather mushrooms every day? -Where do you gather mushrooms? -Do you gather anything else? -Who buys you the mushrooms? -Why don't all mushrooms sellers gather them?

*Questions were not asked literally, but rather were adapted according to the context and circumstances of interviews.

We followed the criteria used to define the mycophily or mycophoby of informants reported previously [3,11], this was through the understanding of the informants TMK and attitudes toward the mushrooms.

Our economic dynamics analysis was performed according to grounded theory [41] and we repeatedly categorized and compared data. Some processes were explained using an economic anthropology substantivist approach [42]. We used concepts as: exchange, redistribution and reciprocity. Exchange is defined by the extra-economic benefits from which a series of social relationships are constructed [43]. Redistribution points out the movements of appropriation to a center and then to the exterior. Reciprocity is the return of a material gift induced by social obligations, typically from kinship. For Sahlins [44], reciprocity is divided into: general reciprocity, where material return is improbable, implicit and voluntary; balanced reciprocity, when material return is immediate and is equal to the received gift value; negative reciprocity, where someone wants to obtain something free, both participants have different interests and someone takes advantage from the other.

## Results and discusion

**Figure 2:**
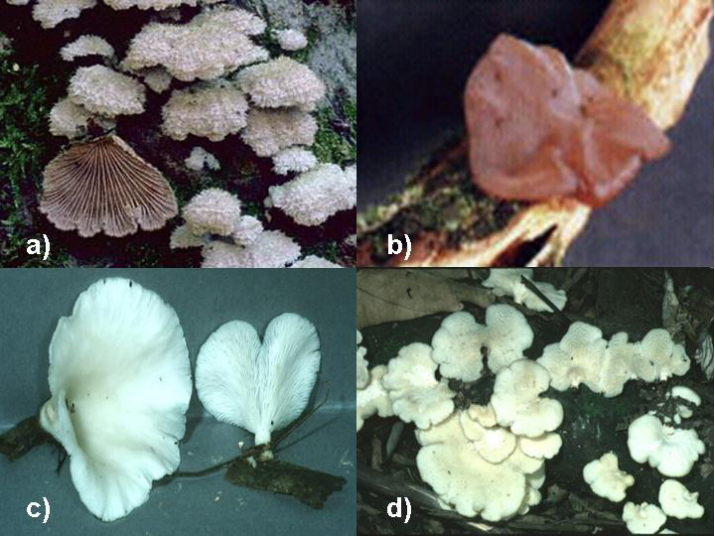
**Mushroom species sold and/or consumed. **a) *Schizophyllum commune*, b) *Auricularia delicata*, c) *Pleurotus djamor*, d)* Favolus tenuiculus*.

We found that *Schizophyllum commune *was sold in four markets of Oaxaca and two markets of Tabasco; *Polyporus tenuiculus *was sold in two markets of Oaxaca; and local consumption of *Auricularia polytricha*, *A. delicata *and *Pleurotus djamor *was present mainly in Tabasco (Figure 2). Table 4 shows where mushrooms were sold and used. The two *Auricularia *species were also used as toys in Tabasco. Children make a small hole in one of the mushroom's membranes and blow inside it to form a little balloon. All species were saprophytic, developed on lignicolous substrates and had a gristly, rubbery or corky consistence. Van Dijk et al. [5] explain the preference for species with a resistant and gristly consistence, which are also found within the inhabitants of the Cameroon rain forest, by the high humidity and temperature conditions of the jungle. Under these conditions, the fruit bodies of etcomycorrizic mushrooms (which are generally fleshier) rot quicker, rendering their transportation, consumption or selling as a much more complicated event.

**Table 4:** Sell, use and species characteristics

Species	Sell	Cws	Use	Consistence	Habit
*Schizophyllum commune*	Tu, Oj, Va, Chi, Te, Ma		E	Corky	Saprobious
*Favolus tenuiculus*	Tu, Oj		E	Rubbery	Saprobious
*Pleurotus djamor*		Cat, Te	E	Rubbery	Saprobious
*Auricularia polytricha*		Te	E, L	Gristly	Saprobious
*Auricularia delicata*		Te	E, L	Gristly	Saprobious

In Sell, Tu: Tuxtepec, Oj: Ojitlán, Va: Valle nacional, Chi: Chiltepec, Te: Teapa, Ma: Macuspana. Csw: Consumption without sell; Cat: Catemaco, Te: Teapa. In Use, E: edible L: ludic.

### Wild mushroom selling dynamics

From the 25 markets visited, 12 were established markets and 13 mobile markets. Out of the 291 interviewees, 22 sold mushrooms. In Table 5 we summarize the types of markets where mushrooms were sold as well as provide a breakdown of ethnic group, gender and habitat of mushroom sellers.

**Table 5:** Sell point's and wild mushroom sellers characteristics

State	Veracruz			Oaxaca					Tabasco			
Settlement	Sant	ST	Cat	Tu	Oj	Vall	Chi	Lo	Vi	Te	Mac	Hui

E. market	0	0	0	0	0	0	0	0	0	0	0	0
M. market	0	0	?	1	1	1	1	0	0	1	1	0
N° of sellers	0	0	0	4	2	3	1	0	0	9	3	0
Men	0	0	0	0	0	0	0	0	0	3	1	0
Women	0	0	0	4	2	3	1	0	0	6	2	0
Ethnic group	-	-	-	Ch	Ch	Ch	Ch	-	-	7 M, 2Z	1Chontal, 2 Ch'ol	-
Habitat	-	-	-	R	R	R	R	-	-	R	R	-

In Settlement, Sant: Santiago Tuxtla, ST: San Andrés Tuxtla, Cat: Catemaco, Tu: Tuxtepec, Oj: Ojitlán, Vall: Valle Nacional, Chi: Chiltepec, Lo: Loma Bonita, Vi: Villahermosa, Te: Teapa, Mac: Macuspana, Hui: Huimanguillo. E. market: Established market; number of markets with wild mushrooms sell. M. market: Mobile market; number of markets with wild mushrooms sell; ?: informants reported mushrooms sell but not verified. In Ethnic group, Ch: Chinantecos, M: Mestizos, Z: Zoques. In Habitat, R: Rural.

### Markets and types of stands

Mushroom selling was present in six mobile markets, four in Oaxaca and two in Tabasco (Table 5). In Catemaco, Veracruz, informants reported the consumption of *Pleurotus djamor *but we were unable to verify it. We also did not observe any wild mushroom being sold in established markets. This is because mobile markets tend to group and sell the products of nearby rural towns. On the other hand, in established markets edible products always came from intermediary chains. In the mobile markets of Loma Bonita, Huimanguillo and in those of Veracruz, no wild mushroom selling was observed. In the first two markets this was because indigenous sellers were not present and in fact, the presence of peasant stands was poor. In all mobile markets visited in Veracruz, the sellers with peasant stands were immigrants established since 1950's and their use of wild edibles is scarce.

### Gender patterns

Women comprised the majority of the sellers (81.82%); in Oaxaca the figure was 100% and in Tabasco 67% (Table 5). The majority of the observed men engaged in selling were there on an occasional basis. In Mexico, some authors [24,45] have reported the same phenomena in Papantla and Poza Rica, Veracruz, and in Toluca respectively. Oso [13] and Prance [22] also report a predominance of women in mushroom gathering and selling in Nigeria and the Brazilian Amazon respectively. According to Prance, since women are more engaged in the gathering of wild edibles, they are the ones who know about mushrooms, while the gender role of men takes them into the jungle as hunters. In our case, we can see a similar pattern emerge. However, it is not a result of differences in traditional knowledge; it is a result of work division: with men dedicating most of their time in the fields and women selling the products.

### Ethnic groups and habitat

In general, the selling of mushrooms was restricted to indigenous groups: Chinantecs in Oaxaca (100%); and Chontales and Ch'oles in Macuspana (100%). Mariaca et al. [45] have mentioned that indigenous people in Mexico have a deeper TMK and also found an intensive use of this resource. The exception was Teapa, where 78% of sellers were mestizos and 22% were Zoques (Table 4). Mestizos from this town have a wide TMK resulting from the high cultural influence received from Zoques from northern Chiapas.

Every mushroom seller lives or has lived in "rancherías" (groups of no more than ten houses far from towns) in intimate contact with nature. Nevertheless, in Veracruz 44% of interviewees lived in rural areas but did not sell mushrooms. This was because, similar to the case of Loma Bonita and Huimanguillo (where mushrooms were also not sold) all sellers were mestizos. Moreover, Veracruz sellers had a limited environmental knowledge given their recent history as immigrants and colonizers.

### Mushroomer presence and mushroom perception

**Figure 3:**
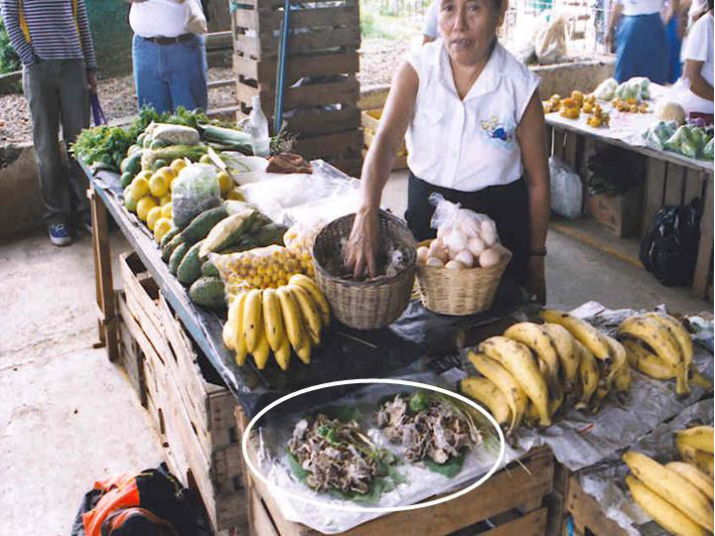
Typical wild mushroom stand in Mexican tropics, mushroom are signaled by an ellipse.

**Figure 4:**
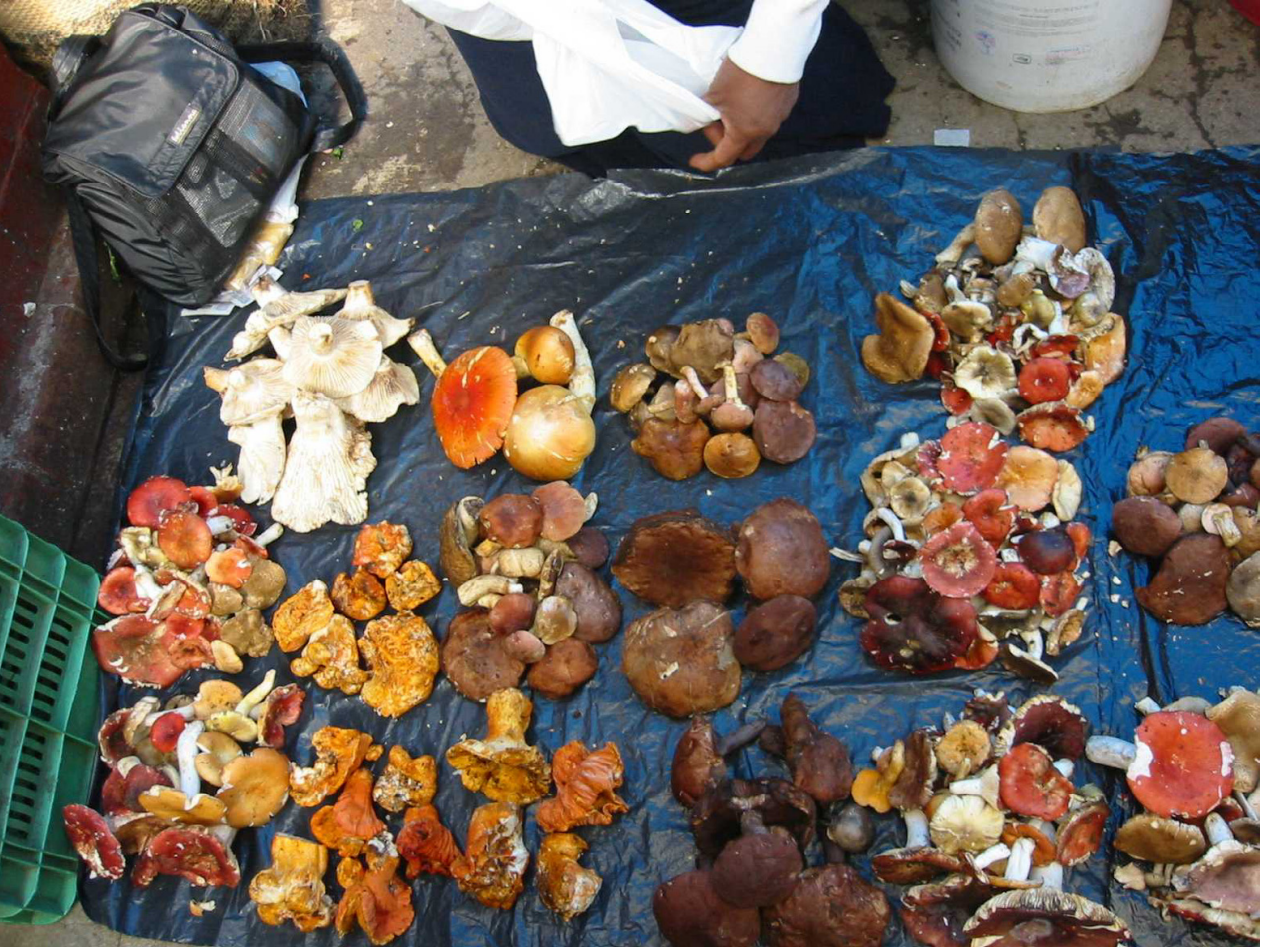
Typical wild mushroom stand in Temperate Mexico.

In every stand where wild mushroom were sold, there was also commercialization of other wild or cultivated vegetal products. Some of these were: "nopales" (*Opuntia ficus-indica*), "acuyo" (*Piper *spp.), "hierba mora" (*Solanum *spp.), "nanches" (*Byrsonima crassifolia*), "tomatillos" (*Physalis *spp.), banana (*Musa paradisiaca *and *M. cavedendishii*), chili (*Capsicum *spp.), orange (*Citrus sinensis*), "epazote" (*Teloxys *spp.), domestic bird eggs, etc. (Figure 3). Mushrooms were perceived as one of the "ranch foods" (ranch here is a generic term used by people to designate every rural area in contact with wild nature). In temperate Mexico, on the other hand, mushrooms are sold alone or with few other products like firewood or coal [45,46] (Figure 4).

People recognized those who sell mushrooms and other products as "ranch product sellers". Thus, mushrooms were not regarded as something special, but instead just one more of the group of traditionally cultivated or wild products. By no means did mushroom sellers recognize themselves as "Hongueros" (Mushroomers), nor were they aware of the existence of "towns of Mushroomers". This contrasts with temperate Mexico where mushrooms sellers call themselves "Hongueros" and recognize "towns of Mushroomers" due to the high number of families involved in their harvesting and selling [45,46].

### Stand dynamics, prices and selling volumes

Gatherers usually bring to the market considerable amounts of *S. commune*, from 45 × 45 cm nylon bags up to large sugar sacks full of it. Guzmán [2] and Sommerkamp [23] have described a similar phenomenon in Petén and Cobán markets in north Guatemala. In Mexico, the consumption of *S. commune *has also been reported in the Mazatec Range and the "Costa Chica" in Oaxaca [47] and in Quintana Roo [48].

**Figure 5:**
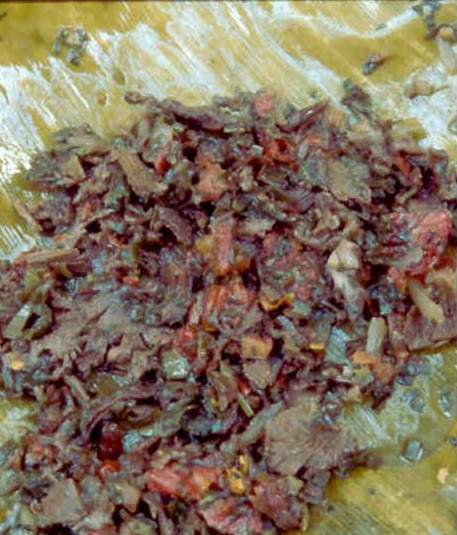
"Mone" a traditional meal made with mushrooms.

Mushrooms were packaged in nylon bags or wrapped in banana leaves. In Teapa there were wrapped in a "momo" leaf (*Piper auritum*) with "cebollín" (*Allium *sp.) and sweet chili (*Capsicum *sp.) (Figure 3). This package contained the basic ingredients to prepare a traditional local meal named "mone" (Figure 5). Each pack had approximately 200 gr. humid-weight and was sold for $0.50 (USD) in Tuxtepec and for $1.00 in Tabasco, i.e. from $2.5 to $5.0/Kg. Resellers kept a reserve amount of mushrooms to prepare more packages if necessary. Stands were installed since sunrise and mushrooms were commonly sold by 10:00 hrs. The merchandise was offered over wood boxes or over plastic carpets on the floor. When mushrooms were not sold, the seller's family consumes them. It was common to observe people buying up to $20.0 of *S. commune *to take it to their places of origin. In Teapa and Tuxtepec there are small restaurants located inside the local markets that offer *S. commune *as a basic part of some of the meals but was most commonly used to prepare "mone" or a local variant of it.

The dynamics of selling *P. tenuiculus *were similar, but the volume was considerably less since stands never offer more than 1 Kg of this mushroom. In Tuxtepec and Ojitlán, mushroom packs contained approximately 300 gr. and cost $0.5, or $1.67/Kg. Mushroom stands were never grouped in any one section of the markets.

Considerable differences exist between these dynamics and those reported in temperate zones in Mexico. There, mushrooms are presented in one or more species piles [46]. Piles are offered over plastics, baskets and trays (Figure 4). Generally, mushrooms stands are located in a specific area outside the market where most Mushroomers offer their products. Prices are contrasting too, nowadays in Tenancingo, Estado de México, cheaper mushrooms (*Russula *spp., *Suillus *spp., etc.) are sold at $3.0/Kg., others like *Lyophyllum *spp. at $5.0/Kg and some *Ramaria *species at $6.0/Kg While in Mexico City mobile markets, *Lyophyllum *spp. is sold from $6.0 to $8.0/Kg. and *Morchella *spp. reaches $20.0/Kg. (personal observations).

### Gathering – selling process

Collectors live in "rancherías" close to the markets. Although we did gathering trips mainly with adult men; boys, girls, and women were involved actively in mushroom gathering too. In interviews, women reported to go alone or with children to harvest wild edibles including mushrooms. However, we could not see this because men never allowed us to harvest mushrooms alone with their women. The harvest is done in the same sites where people work, given that their main activities are agriculture and cattle raising. Thus casual gathering did not represent an additional waste of time or energy. Zent et al. [8] report the same situation among "Hotï" in the Venezuelan Amazon, where mushroom gathering is basically an opportunistic activity.

People sold mushrooms one day after they were gathered. They did not have a specific day or periodicity for selling them. This again contrasts with temperate zones were Mushroomers undertake long journeys dedicating anywhere from five to eight hours with the sole objective of gathering mushrooms; in fact, mushroomers can make one or two trips per day in order to maximize the amount of mushrooms they collect [45,46].

Eighty-two percent of mushroom sellers did not collect mushrooms themselves. In Oaxaca, no sellers where involved in the gathering process. Sellers from Tuxtepec bought fungi and other edible vegetables in Ojitlán. In Tabasco and Ojitlán, gatherers sold their products directly to stand owners who then resold the mushrooms. These gatherers did not recognize themselves as Mushroomers. This is in sharp contrast to what happens in temperate Mexico, where mushroom sellers, principally in secondary markets, are also gatherers [45,46].

Even those sellers living in rural areas depend on intermediaries, since gaining access to places where mushrooms grow is not an easy task. Mushrooms are gathered in corn fields, secondary vegetation and cattle fields where fallen trunks are abundant. Few sellers own land with these characteristics, resulting in only a few people being able to exploit the resource. In Teapa and Macuspana, we found four collectors (two in each town) directly selling their products. In these cases, collectors, their families or friends own land that is conducive to gathering. Therefore, we found that mushroom selling was always a function of land access. Mariaca, et al. [45] reports that in temperate woodlands mushroomers do not respect properties or borders, nor he saw evidence of owners deterring illegal entries to their lands. However, in these areas gathering takes place mainly in the woods [46,49] whereas in tropical areas gathering is done in privately owned land parcels that are exploited by their owners only. Härkönen et al. [14] also reports that in most parts of Tanzania everyone is free to collect mushrooms virtually anywhere.

### Economic anthropology notes

Most mushroom sellers obtained an income of up to 100% of the invested capital. However, in Tuxtepec, we observed an additional pattern. There, the profit obtained by mushroom selling was minimum or inexistent. Based on our observations, this can be explained as a form of exchange. In this sense, mushrooms were transported to Tuxtepec by sellers, bringing them to city Chinantecs who fully appreciate the product. The Chinantecs in turn, when in need of supplies such as wild plants buy them from stalls that sell mushrooms. They do this out of a sense of "moral obligation" and thus apart from buying mushrooms they also buy others supplies offered in these stalls as well. Because wild plants comprise the main proportion of sale revenue in these stalls, a cero gain in mushroom selling was not negative. Polanyi's [42] concept of reciprocity is also useful here to explain this relation, pointing out the movements between symmetrical elements.

Mushroom gatherers occasionally brought mushrooms as gifts for relatives and friends (typically from Tuxtepec). In this case, a bartering system as described by Casaverde [43] was established. The visitor brings rural products to their hosts, thus enhancing social relationships between them. This ensures that in reciprocal visits there is a flow of products from urban centers to the rural settlements inside the forest. In all cases, the logic among participants was the improvement of their social relationships and not just a capitalist commodification of mushrooms. Garibay-Orijel et al. [47] have described a similar relation of reciprocal gifting among Zapotecs of Oaxaca highlands.

### Mycophily and mycophoby inferences

From the total of interviewees, Spanish speaking people from urban areas (60% of informants), didn't provide a lot of information when asked about mushrooms or their reasons for being in the market. In fact they showed certain mistrust talking about the topic, showing no interest and even dislike for mushrooms. On the other hand, Spanish speakers from rural areas (30% of informants) possessed more mushroom related knowledge although they did not particularly appreciated them as food product. However, indigenous inhabitants of rural areas (10% of informants) showed a more detailed local mycological knowledge as well as a wider acceptance as an edible resource. In contrast with the observations from studies by Mapes et al. [4] and Goes-Neto and Bandeira [3], here we cannot observe a widespread mycophoby or non-mycophily among inhabitants of tropical areas but rather a differential sympathy related to ethnic origin and habitat conditions. Spanish speaker informants from urban areas can be classified as mycophobic while rural Spanish speaker informants can be classified as non-mycophobic. Although most of the interviewees were not mycophilic, a percentage of them were truly mycophilic: and they all shared the fact of being indigenous and inhabitants of rural areas.

## Conclusion

In the studied area, just *S. commune *and *P. tenuiculus *were sold, and the main contrast with other fleshy species of temperate zones is their rubbery consistence.

Mushroom selling happened only in mobile markets and just in peasant stands. Mushroom sellers were mainly women and a considerable amount were indigenous. All they were inhabitants of rural areas. Mushroom sellers did not gather mushrooms themselves because gathering was always restricted by land tenure. It was done mainly in corn fields, cattle fields and secondary vegetation. Gathering was a secondary activity where any family member could participate.

*Schizophyllum commune *had a high cultural value where the mushroom sale occurred; because of its wide presence, total volume of sales and preference within people.

Mushroom sale obviously had a profit-oriented sense. However, among Chinantecs practices such as exchange, reciprocity and barter were common.

There are clear differences among inhabitants of temperate areas and tropical areas concerning their traditional mycological knowledge and practices, as different authors have postulated [4]. However, the sale of these products, the large amounts of commercialization of *S. commune*, the complicated intermediary process, as well as the insertion of mushrooms into different informal economic practices are all evidence of an existent mycophily in a sector of the population of this region of the Mexican tropics. Within our sample, urban mestizo people were mycophobic, rural mestizo people were non-mycophilic and indigenous people were mycophilic.

## Authors' contributions

FR designed the study, carried out the field work, the analysis and interpretation of data and drafted the manuscript. RG participated in the design of the study, in the analysis and interpretation of data and helped to draft the final manuscript. JC collaborated in the taxonomic determination of the mycological material, made substantial contributions to the analysis and the revision of the document. All authors read and approved the final manuscript.
